# Bis[4-hydr­oxy-*N*′-(4-meth­oxy-2-oxido­benzyl­idene-κ*O* ^2^)benzohydrazidato-κ^2^ *O*,*N*′]cadmium(II) dimethyl sulfoxide disolvate

**DOI:** 10.1107/S1600536809013774

**Published:** 2009-04-22

**Authors:** Nooraziah Mohd Lair, Hapipah Mohd Ali, Seik Weng Ng

**Affiliations:** a Department of Chemistry, University of Malaya, 50603 Kuala Lumpur, Malaysia

## Abstract

The metal atom in the title compound, [Cd(C_15_H_13_N_2_O_4_)_2_]·2C_2_H_6_OS, is twice *O*,*N*,*O*′-chelated by two symmetry-related Schiff base ligands to define a *trans*-N_2_O_4_ octa­hedral geometry. Each anion occupies meridional sites of the octa­hedron; the metal atom lies on a special position of site symmetry 2. The dimethyl sulfoxide mol­ecule is a hydrogen-bond acceptor to the –NH– unit, and O—H⋯O hydrogen bonds link mol­ecules into a supra­molecular chain.

## Related literature

For the monohydrated Schiff base ligand, see: Mohd Lair *et al.* (2009 ▶).
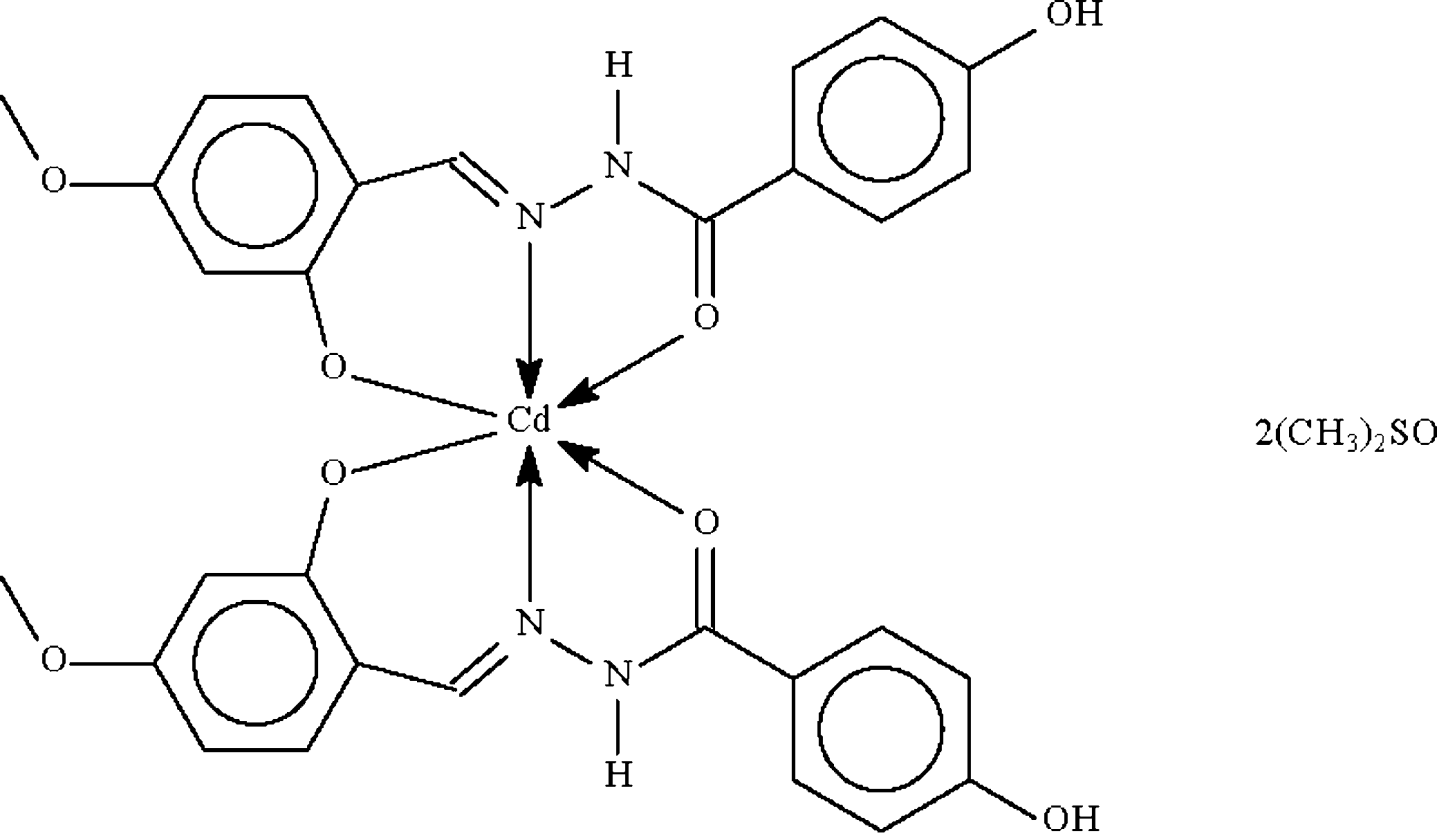


## Experimental

### 

#### Crystal data

[Cd(C_15_H_13_N_2_O_4_)_2_]·2C_2_H_6_OS
*M* *_r_* = 839.20
Monoclinic, 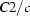

*a* = 23.891 (2) Å
*b* = 10.439 (1) Å
*c* = 19.874 (1) Å
β = 132.137 (4)°
*V* = 3675.3 (4) Å^3^
*Z* = 4
Mo *K*α radiation
μ = 0.77 mm^−1^
*T* = 118 K
0.12 × 0.06 × 0.03 mm

#### Data collection

Bruker SMART APEX diffractometer
Absorption correction: multi-scan (*SADABS*; Sheldrick, 1996 ▶) *T*_min_ = 0.507, *T*_max_ = 0.745 (expected range = 0.665–0.977)
10208 measured reflections
3243 independent reflections
2147 reflections with *I* > 2σ(*I*)
*R*_int_ = 0.103

#### Refinement

*R*[*F* ^2^ > 2σ(*F* ^2^)] = 0.059
*wR*(*F* ^2^) = 0.153
*S* = 1.02
3243 reflections
211 parameters
H-atom parameters constrained
Δρ_max_ = 1.31 e Å^−3^
Δρ_min_ = −0.90 e Å^−3^

### 

Data collection: *APEX2* (Bruker, 2008 ▶); cell refinement: *SAINT* (Bruker, 2008 ▶); data reduction: *SAINT*; program(s) used to solve structure: *SHELXS97* (Sheldrick, 2008 ▶); program(s) used to refine structure: *SHELXL97* (Sheldrick, 2008 ▶); molecular graphics: *X-SEED* (Barbour, 2001 ▶); software used to prepare material for publication: *publCIF* (Westrip, 2009 ▶).

## Supplementary Material

Crystal structure: contains datablocks global, I. DOI: 10.1107/S1600536809013774/tk2421sup1.cif

Structure factors: contains datablocks I. DOI: 10.1107/S1600536809013774/tk2421Isup2.hkl

Additional supplementary materials:  crystallographic information; 3D view; checkCIF report

## Figures and Tables

**Table 1:** Hydrogen-bond geometry (Å, °)

*D*—H⋯*A*	*D*—H	H⋯*A*	*D*⋯*A*	*D*—H⋯*A*
O4—H4⋯O1^i^	0.84	1.79	2.603 (6)	163
N2—H2⋯O5	0.88	1.93	2.766 (6)	159

Symmetry code: (i) 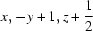.

**Fig. 1.**
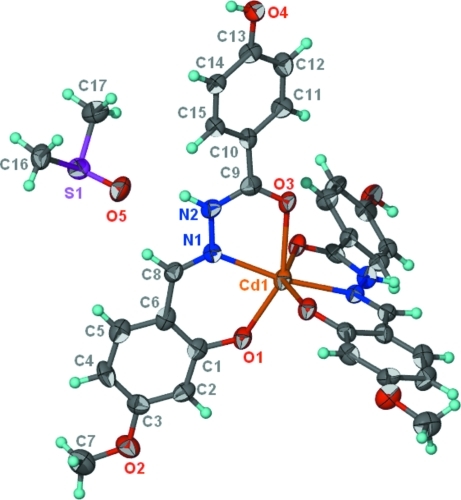
Thermal ellipsoid plot (Barbour, 2001) of Cd(C15H13N2O4)2.2DMSO at the 70% probability level. Hydrogen atoms are drawn as spheres of arbitrary radius.

## supplementary crystallographic information

### Experimental

4-Hydroxy-*N*'-(2-hydroxy-4-methoxybenzylidene)benzohydrazide monohydrate (0.30 g, 1 mmol) and cadmium diacetate (0.14 g, 0.5 mmol) were heated in ethanol (50 ml) for 4 h. The solvent was removed and the product was recrystallized from DMSO to give prismatic crystals.

### Refinement

Owing to the small number of observed reflections, the aromatic rings were refined as rigid hexagons with sides of 1.39 Å in order to reduce the number of refined parameters. Hydrogen atoms were placed at calculated positions (C–H 0.95–0.98, N–H 0.88, O–H 0.84 Å) and were treated as riding on their parent carbon atoms, with *U*(H) set to 1.2–1.5 times *U*_eq_(*C*,*N*,*O*). The final difference Fourier map had a large peak/deep hole in the vicinity of the Cd atom.

### Crystal data

**Table:**

[Cd(C_15_H_13_N_2_O_4_)_2_]·2C_2_H_6_OS	*F*(000) = 1720
*M**_r_* = 839.20	*D*_x_ = 1.517 Mg m^−^^3^
Monoclinic, *C*2/*c*	Mo *K*α radiation, λ = 0.71073 Å
Hall symbol: -C 2yc	Cell parameters from 638 reflections
*a* = 23.891 (2) Å	θ = 2.2–18.8°
*b* = 10.439 (1) Å	µ = 0.77 mm^−^^1^
*c* = 19.874 (1) Å	*T* = 118 K
β = 132.137 (4)°	Prism, yellow
*V* = 3675.3 (4) Å^3^	0.12 × 0.06 × 0.03 mm
*Z* = 4	

### Data collection

**Table:**

Bruker SMART APEX diffractometer	3243 independent reflections
Radiation source: fine-focus sealed tube	2147 reflections with *I* > 2σ(*I*)
graphite	*R*_int_ = 0.103
ω scans	θ_max_ = 25.0°, θ_min_ = 2.1°
Absorption correction: multi-scan (*SADABS*; Sheldrick, 1996)	*h* = −28→28
*T*_min_ = 0.507, *T*_max_ = 0.745	*k* = −12→12
10208 measured reflections	*l* = −23→23

### Refinement

**Table:**

Refinement on *F*^2^	Primary atom site location: structure-invariant direct methods
Least-squares matrix: full	Secondary atom site location: difference Fourier map
*R*[*F*^2^ > 2σ(*F*^2^)] = 0.059	Hydrogen site location: inferred from neighbouring sites
*wR*(*F*^2^) = 0.153	H-atom parameters constrained
*S* = 1.02	*w* = 1/[σ^2^(*F*_o_^2^) + (0.0692*P*)^2^ + 0.0136*P*] where *P* = (*F*_o_^2^ + 2*F*_c_^2^)/3
3243 reflections	(Δ/σ)_max_ = 0.001
211 parameters	Δρ_max_ = 1.31 e Å^−^^3^
0 restraints	Δρ_min_ = −0.90 e Å^−^^3^

### Fractional atomic coordinates and isotropic or equivalent isotropic displacement parameters (Å^2^)

**Table:**

	*x*	*y*	*z*	*U*_iso_*/*U*_eq_	
Cd1	0.5000	0.32500 (7)	0.7500	0.0253 (2)	
S1	0.18289 (10)	0.48574 (18)	0.67220 (12)	0.0335 (5)	
O1	0.4272 (2)	0.1893 (4)	0.6323 (3)	0.0276 (10)	
O2	0.2426 (3)	−0.1133 (5)	0.4273 (3)	0.0373 (12)	
O3	0.5137 (2)	0.4972 (4)	0.8391 (3)	0.0264 (10)	
O4	0.4911 (2)	0.8669 (4)	1.0697 (3)	0.0304 (11)	
H4	0.4624	0.8507	1.0791	0.046*	
N1	0.3944 (3)	0.3437 (5)	0.7273 (3)	0.0227 (12)	
N2	0.3971 (3)	0.4373 (5)	0.7788 (4)	0.0262 (13)	
H2	0.3589	0.4493	0.7755	0.031*	
O5	0.2603 (2)	0.4283 (5)	0.7353 (4)	0.0432 (13)	
C1	0.35890 (17)	0.1366 (4)	0.5922 (3)	0.0261 (16)	
C2	0.3336 (2)	0.0367 (4)	0.5313 (3)	0.0291 (16)	
H2A	0.3643	0.0060	0.5204	0.035*	
C3	0.2633 (2)	−0.0181 (4)	0.4865 (3)	0.0295 (16)	
C4	0.21840 (18)	0.0268 (4)	0.5025 (3)	0.0339 (17)	
H4A	0.1704	−0.0107	0.4719	0.041*	
C5	0.2437 (2)	0.1267 (4)	0.5634 (3)	0.0326 (17)	
H5	0.2130	0.1574	0.5743	0.039*	
C6	0.3140 (2)	0.1815 (4)	0.6082 (3)	0.0286 (15)	
C7	0.1732 (4)	−0.1797 (8)	0.3856 (5)	0.0438 (19)	
H7A	0.1662	−0.2488	0.3471	0.066*	
H7B	0.1309	−0.1196	0.3484	0.066*	
H7C	0.1754	−0.2159	0.4328	0.066*	
C8	0.3314 (4)	0.2851 (6)	0.6694 (5)	0.0269 (16)	
H8	0.2915	0.3119	0.6658	0.032*	
C9	0.4604 (4)	0.5100 (6)	0.8344 (4)	0.0269 (15)	
C10	0.4616 (2)	0.6071 (4)	0.8911 (2)	0.0236 (15)	
C11	0.5075 (2)	0.7141 (4)	0.9198 (3)	0.0289 (16)	
H11	0.5330	0.7276	0.8990	0.035*	
C12	0.5159 (2)	0.8012 (3)	0.9790 (3)	0.0314 (17)	
H12	0.5472	0.8743	0.9986	0.038*	
C13	0.4785 (2)	0.7813 (4)	1.0094 (3)	0.0282 (16)	
C14	0.4326 (2)	0.6744 (4)	0.9807 (3)	0.0241 (14)	
H14	0.4071	0.6609	1.0015	0.029*	
C15	0.4242 (2)	0.5873 (3)	0.9215 (3)	0.0244 (15)	
H15	0.3929	0.5142	0.9019	0.029*	
C16	0.1442 (4)	0.4487 (8)	0.7214 (5)	0.0372 (18)	
H16A	0.1335	0.3567	0.7154	0.056*	
H16B	0.0974	0.4970	0.6902	0.056*	
H16C	0.1805	0.4720	0.7858	0.056*	
C17	0.1980 (4)	0.6515 (7)	0.6951 (5)	0.0424 (19)	
H17A	0.2175	0.6882	0.6690	0.064*	
H17B	0.2346	0.6653	0.7609	0.064*	
H17C	0.1502	0.6932	0.6682	0.064*	

### Atomic displacement parameters (Å^2^)

**Table:**

	*U*^11^	*U*^22^	*U*^33^	*U*^12^	*U*^13^	*U*^23^
Cd1	0.0238 (4)	0.0338 (4)	0.0243 (4)	0.000	0.0186 (3)	0.000
S1	0.0368 (10)	0.0355 (11)	0.0337 (11)	−0.0019 (8)	0.0259 (9)	−0.0040 (9)
O1	0.025 (2)	0.037 (3)	0.027 (3)	0.000 (2)	0.019 (2)	0.000 (2)
O2	0.041 (3)	0.041 (3)	0.032 (3)	−0.010 (2)	0.026 (3)	−0.012 (2)
O3	0.025 (2)	0.035 (3)	0.029 (3)	−0.001 (2)	0.022 (2)	−0.003 (2)
O4	0.034 (3)	0.038 (3)	0.032 (3)	−0.007 (2)	0.028 (2)	−0.010 (2)
N1	0.024 (3)	0.026 (3)	0.020 (3)	−0.001 (2)	0.016 (2)	0.000 (2)
N2	0.030 (3)	0.033 (3)	0.025 (3)	0.000 (3)	0.022 (3)	−0.005 (3)
O5	0.035 (3)	0.042 (3)	0.061 (4)	0.004 (2)	0.036 (3)	0.002 (3)
C1	0.025 (3)	0.031 (4)	0.020 (4)	0.006 (3)	0.014 (3)	0.007 (3)
C2	0.034 (4)	0.033 (4)	0.027 (4)	0.007 (3)	0.024 (3)	0.002 (3)
C3	0.037 (4)	0.025 (4)	0.032 (4)	−0.003 (3)	0.025 (4)	0.000 (3)
C4	0.024 (3)	0.045 (5)	0.021 (4)	−0.012 (3)	0.010 (3)	−0.006 (3)
C5	0.030 (4)	0.044 (5)	0.026 (4)	0.005 (3)	0.019 (3)	0.003 (3)
C6	0.027 (3)	0.035 (4)	0.026 (4)	0.007 (3)	0.018 (3)	0.010 (4)
C7	0.047 (4)	0.044 (5)	0.035 (4)	−0.004 (4)	0.025 (4)	−0.002 (4)
C8	0.027 (4)	0.024 (4)	0.035 (4)	0.003 (3)	0.024 (3)	0.005 (3)
C9	0.027 (4)	0.027 (4)	0.022 (4)	0.007 (3)	0.015 (3)	0.006 (3)
C10	0.021 (3)	0.029 (4)	0.020 (4)	−0.002 (3)	0.014 (3)	0.000 (3)
C11	0.035 (4)	0.034 (4)	0.029 (4)	−0.001 (3)	0.026 (3)	−0.002 (3)
C12	0.033 (4)	0.036 (5)	0.035 (4)	−0.006 (3)	0.027 (3)	0.001 (3)
C13	0.035 (4)	0.033 (4)	0.022 (4)	0.005 (3)	0.021 (3)	0.002 (3)
C14	0.025 (3)	0.029 (4)	0.021 (3)	0.004 (3)	0.017 (3)	0.003 (3)
C15	0.026 (3)	0.026 (4)	0.020 (4)	−0.002 (3)	0.015 (3)	0.001 (3)
C16	0.031 (4)	0.051 (5)	0.037 (5)	−0.004 (3)	0.025 (4)	−0.006 (4)
C17	0.050 (4)	0.039 (5)	0.047 (5)	0.004 (4)	0.035 (4)	−0.001 (4)

### Geometric parameters (Å, °)

**Table:**

Cd1—O1	2.246 (4)	C4—H4A	0.9500
Cd1—O1^i^	2.246 (4)	C5—C6	1.3900
Cd1—N1^i^	2.254 (5)	C5—H5	0.9500
Cd1—N1	2.254 (5)	C6—C8	1.464 (7)
Cd1—O3	2.386 (4)	C7—H7A	0.9800
Cd1—O3^i^	2.386 (4)	C7—H7B	0.9800
S1—O5	1.497 (5)	C7—H7C	0.9800
S1—C17	1.764 (7)	C8—H8	0.9500
S1—C16	1.782 (6)	C9—C10	1.500 (7)
O1—C1	1.362 (5)	C10—C11	1.3900
O2—C3	1.355 (5)	C10—C15	1.3900
O2—C7	1.439 (8)	C11—C12	1.3900
O3—C9	1.221 (7)	C11—H11	0.9500
O4—C13	1.356 (5)	C12—C13	1.3900
O4—H4	0.8400	C12—H12	0.9500
N1—C8	1.280 (8)	C13—C14	1.3900
N1—N2	1.385 (7)	C14—C15	1.3900
N2—C9	1.356 (8)	C14—H14	0.9500
N2—H2	0.8800	C15—H15	0.9500
C1—C2	1.3900	C16—H16A	0.9800
C1—C6	1.3900	C16—H16B	0.9800
C2—C3	1.3900	C16—H16C	0.9800
C2—H2A	0.9500	C17—H17A	0.9800
C3—C4	1.3900	C17—H17B	0.9800
C4—C5	1.3900	C17—H17C	0.9800
			
O1—Cd1—O1^i^	101.8 (2)	C5—C6—C8	112.7 (3)
O1—Cd1—N1^i^	104.12 (16)	C1—C6—C8	127.3 (3)
O1^i^—Cd1—N1^i^	82.26 (16)	O2—C7—H7A	109.5
O1—Cd1—N1	82.26 (16)	O2—C7—H7B	109.5
O1^i^—Cd1—N1	104.12 (16)	H7A—C7—H7B	109.5
N1^i^—Cd1—N1	170.1 (3)	O2—C7—H7C	109.5
O1—Cd1—O3	150.75 (14)	H7A—C7—H7C	109.5
O1^i^—Cd1—O3	94.45 (15)	H7B—C7—H7C	109.5
N1^i^—Cd1—O3	102.05 (16)	N1—C8—C6	127.4 (5)
N1—Cd1—O3	70.17 (16)	N1—C8—H8	116.3
O1—Cd1—O3^i^	94.45 (15)	C6—C8—H8	116.3
O1^i^—Cd1—O3^i^	150.75 (14)	O3—C9—N2	122.3 (6)
N1^i^—Cd1—O3^i^	70.17 (16)	O3—C9—C10	121.5 (6)
N1—Cd1—O3^i^	102.05 (16)	N2—C9—C10	116.1 (5)
O3—Cd1—O3^i^	82.3 (2)	C11—C10—C15	120.0
O5—S1—C17	104.7 (3)	C11—C10—C9	117.7 (3)
O5—S1—C16	104.9 (3)	C15—C10—C9	122.0 (4)
C17—S1—C16	99.3 (4)	C12—C11—C10	120.0
C1—O1—Cd1	130.6 (3)	C12—C11—H11	120.0
C3—O2—C7	117.6 (5)	C10—C11—H11	120.0
C9—O3—Cd1	114.2 (4)	C11—C12—C13	120.0
C13—O4—H4	109.5	C11—C12—H12	120.0
C8—N1—N2	116.5 (5)	C13—C12—H12	120.0
C8—N1—Cd1	128.4 (4)	O4—C13—C14	122.6 (3)
N2—N1—Cd1	114.9 (3)	O4—C13—C12	117.3 (3)
C9—N2—N1	118.2 (5)	C14—C13—C12	120.0
C9—N2—H2	120.9	C13—C14—C15	120.0
N1—N2—H2	120.9	C13—C14—H14	120.0
O1—C1—C2	117.6 (3)	C15—C14—H14	120.0
O1—C1—C6	122.4 (3)	C14—C15—C10	120.0
C2—C1—C6	120.0	C14—C15—H15	120.0
C1—C2—C3	120.0	C10—C15—H15	120.0
C1—C2—H2A	120.0	S1—C16—H16A	109.5
C3—C2—H2A	120.0	S1—C16—H16B	109.5
O2—C3—C2	116.1 (3)	H16A—C16—H16B	109.5
O2—C3—C4	123.9 (3)	S1—C16—H16C	109.5
C2—C3—C4	120.0	H16A—C16—H16C	109.5
C5—C4—C3	120.0	H16B—C16—H16C	109.5
C5—C4—H4A	120.0	S1—C17—H17A	109.5
C3—C4—H4A	120.0	S1—C17—H17B	109.5
C4—C5—C6	120.0	H17A—C17—H17B	109.5
C4—C5—H5	120.0	S1—C17—H17C	109.5
C6—C5—H5	120.0	H17A—C17—H17C	109.5
C5—C6—C1	120.0	H17B—C17—H17C	109.5
			
O1^i^—Cd1—O1—C1	90.7 (4)	C3—C4—C5—C6	0.0
N1^i^—Cd1—O1—C1	175.5 (4)	C4—C5—C6—C1	0.0
N1—Cd1—O1—C1	−12.2 (4)	C4—C5—C6—C8	180.0 (4)
O3—Cd1—O1—C1	−31.7 (6)	O1—C1—C6—C5	178.1 (4)
O3^i^—Cd1—O1—C1	−113.8 (4)	C2—C1—C6—C5	0.0
O1—Cd1—O3—C9	24.1 (6)	O1—C1—C6—C8	−1.8 (6)
O1^i^—Cd1—O3—C9	−99.9 (4)	C2—C1—C6—C8	−179.9 (5)
N1^i^—Cd1—O3—C9	177.1 (4)	N2—N1—C8—C6	−178.8 (5)
N1—Cd1—O3—C9	3.5 (4)	Cd1—N1—C8—C6	6.1 (9)
O3^i^—Cd1—O3—C9	109.4 (5)	C5—C6—C8—N1	171.3 (6)
O1—Cd1—N1—C8	2.3 (5)	C1—C6—C8—N1	−8.8 (9)
O1^i^—Cd1—N1—C8	−97.9 (5)	Cd1—O3—C9—N2	−3.9 (8)
O3—Cd1—N1—C8	172.4 (6)	Cd1—O3—C9—C10	175.5 (4)
O3^i^—Cd1—N1—C8	95.3 (5)	N1—N2—C9—O3	1.4 (9)
O1—Cd1—N1—N2	−172.8 (4)	N1—N2—C9—C10	−178.1 (5)
O1^i^—Cd1—N1—N2	86.9 (4)	O3—C9—C10—C11	25.7 (7)
O3—Cd1—N1—N2	−2.8 (4)	N2—C9—C10—C11	−154.8 (4)
O3^i^—Cd1—N1—N2	−79.9 (4)	O3—C9—C10—C15	−148.5 (5)
C8—N1—N2—C9	−173.7 (6)	N2—C9—C10—C15	31.0 (7)
Cd1—N1—N2—C9	2.1 (7)	C15—C10—C11—C12	0.0
Cd1—O1—C1—C2	−168.0 (3)	C9—C10—C11—C12	−174.3 (4)
Cd1—O1—C1—C6	13.8 (6)	C10—C11—C12—C13	0.0
O1—C1—C2—C3	−178.2 (4)	C11—C12—C13—O4	177.6 (4)
C6—C1—C2—C3	0.0	C11—C12—C13—C14	0.0
C7—O2—C3—C2	174.7 (5)	O4—C13—C14—C15	−177.4 (4)
C7—O2—C3—C4	−6.5 (7)	C12—C13—C14—C15	0.0
C1—C2—C3—O2	178.9 (5)	C13—C14—C15—C10	0.0
C1—C2—C3—C4	0.0	C11—C10—C15—C14	0.0
O2—C3—C4—C5	−178.8 (5)	C9—C10—C15—C14	174.1 (4)
C2—C3—C4—C5	0.0		

Symmetry codes: (i) −*x*+1, *y*, −*z*+3/2.

### Hydrogen-bond geometry (Å, °)

**Table:**

*D*—H···*A*	*D*—H	H···*A*	*D*···*A*	*D*—H···*A*
O4—H4···O1^ii^	0.84	1.79	2.603 (6)	163
N2—H2···O5	0.88	1.93	2.766 (6)	159

Symmetry codes: (ii) *x*, −*y*+1, *z*+1/2.
